# Fluid dynamics and epidemiology: Seasonality and transmission dynamics

**DOI:** 10.1063/5.0037640

**Published:** 2021-02-02

**Authors:** Talib Dbouk, Dimitris Drikakis

**Affiliations:** University of Nicosia, Nicosia CY-2417, Cyprus

## Abstract

Epidemic models do not account for the effects of climate conditions on the transmission dynamics of viruses. This study presents the vital relationship between weather seasonality, airborne virus transmission, and pandemic outbreaks over a whole year. Using the data obtained from high-fidelity multi-phase, fluid dynamics simulations, we calculate the concentration rate of Coronavirus particles in contaminated saliva droplets and use it to derive a new Airborne Infection Rate (AIR) index. Combining the simplest form of an epidemiological model, the susceptible–infected–recovered, and the AIR index, we show through data evidence how weather seasonality induces two outbreaks per year, as it is observed with the COVID-19 pandemic worldwide. We present the results for the number of cases and transmission rates for three cities, New York, Paris, and Rio de Janeiro. The results suggest that two pandemic outbreaks per year are inevitable because they are directly linked to what we call weather seasonality. The pandemic outbreaks are associated with changes in temperature, relative humidity, and wind speed independently of the particular season. We propose that epidemiological models must incorporate climate effects through the AIR index.

## I. INTRODUCTION

COVID-19 witnessed since late 2019^1^ is one of the largest health and economic crisis events in current history. Governmental institutions and political organizations have encountered various challenges in managing the complications the pandemic arose. To delay the acceleration of COVID-19’s airborne virus transmission, governments introduced general lockdown strategies following advice by scientists. The increasing number of daily infections could result in public health systems being unable to accommodate all patients for treatment and recovery. Thus, governments extended restrictions to encompass social concerns, e.g., use of face masks and traveling restrictions.^2^ These solutions are useful only in slowing the pace of the total number of newly infected individuals.^3^ The above helps absorb the shock wave of the pandemic outbreak and, more importantly, to avoid the saturation of hospitals and emergency centers.

To implement extensive scale restrictions, governments often consult predictive mathematical models (stochastic or deterministic) to forecast the new number of infected individuals that might occur in the short-term (e.g., one or two weeks). Thus, pandemic simulations guide and shape the national political responses and decisions^4,5^ over a short period. The official number of the daily infected cases announced by federal authorities is doubtful. It does not represent the real number as it is merely dependant on the number of tests performed, failing to consider the number of non-tested individuals. The above implies that all pandemic predictive models are inaccurate, but some might be useful.^6^ Despite inaccuracies, this general estimation can be utilized to predict a new number of cases subject to the same scale of the announced data being used to fit some part of the model. The above is like expecting the next single point at the extremity of a smooth curve that is made out of thousands of points. Therefore, most published pandemic prediction models in the literature could be useful, although none can forecast the shape of a pandemic curve over a long period, e.g., a year.

The most reliable models are not necessarily the most complex ones but are those that contain fewer parameters. This is because the computational uncertainty, and, in turn, the prediction error, generally decreases with fewer model parameters. Past studies highlight^7^ the facts behind the unreasonable effectiveness of simple models when applied to the COVID-19 pandemic, such as the SIR (Susceptible–Infected–Recovered) model,^8^ which is the early basic simple model used for epidemic modeling and prediction. The significant advantage behind the basic SIR model is that it contains only two parameters: a transmission rate (*β*) and a recovery rate (*γ*). These parameters represent the probability per unit time that a susceptible individual becomes infected and the probability per unit time that an infected person becomes recovered and immunized.

Scientists applied the SIR and its extensions to predict pandemic and epidemic outbreaks in different types of disease propagation.^6^ They usually fit their model with the available data and then indicate the number of new cases at the extremity of a short period, e.g., a few days or a couple of weeks. The pandemic forecasting, however, is a highly nonlinear process, and epidemiological models try to examine the implications of different parameters for which we have insufficient knowledge of their intertwining effects. Models try to take into account various social contacting behaviors to adapt their structure to the available data.

Unfortunately, none of the existing pandemic models (SIR or SIR-derived) attempts to link the transmission rate to the climate conditions, i.e., a physics-based transmission rate parameter in the SIR model. Similarly, none of the SIR-derived models linked the recovery rate to the number of days required biologically for an ailing individual to recover. Usually, healthy people with no specific diseases need three to five days, thus a recovery rate of *γ* ∈ [1/3–1/5] days^−1^. Older adults or individuals with health problems (diabetes, heart, etc.) need ten days or more to recover; thus, *γ* < 1/14 days^−1^.

When a subject speaks, coughs, or sneezes (hereafter called the “event”), they emit human mucus and saliva droplets into the environment. The above is directly linked to the transmission of infectious diseases like the COVID-19. Fluid dynamics is an ancient scientific discipline, but it is also an emerging discipline when applied to our understanding of airborne disease transmission.^9^ Computational Fluid Dynamics (CFD) constitutes an advanced modeling approach, which allows the study of flow, heat, and mass transport of airborne virus transmission in a physics-based simulation framework. One cannot study the complex multi-phase fluid dynamics phenomena of airborne transmission *in vivo* due to instrumentation and time constraints.^10^ Several scientific studies have emerged aiming at increasing our understanding of different phenomena related to the CoV.^10–16^

Previously, we have performed extensive multi-physics CFD simulations to investigate the phenomena of contaminated saliva droplets transport after being expelled from an infected individual’s mouth into the environment.^10,11^ Furthermore, we have carried detailed studies of a broad range of weather conditions, i.e., temperature T = [0 °C, 10 °C, 20 °C, 30 °C, 40 °C], wind speed U_W*ind*_ = [4 km/h, 10 km/h, 15 km/h, 20 km/h], and relative humidity (RH) = [10%, 30%, 50%, 70%, 100%].

Using the fluid dynamics and heat transfer findings of Dbouk and Drikakis,^10,11,17^ here we develop new models for the epidemiological dynamics. We compute the concentration *C* = *C*_*CoV*_ of CoV particles in contaminated saliva droplets (suspended in the air) and the concentration rate (*CR*) as a function of *T*, *U*_w*ind*_, and *RH*. The concentration rate *∂*(*C*/*C*_0_)/*∂t* is found to be negative in its sign, thus indicating that it decreases with the increase in temperature and increases with the increase in both relative humidity and wind speed. Therefore, CR represents an excellent indicator for quantifying, what we introduce in this study, a weather-dependent Airborne Infection Rate (*AIR*) index (*AIR* = *CR*). AIR indicates the viability of the airborne virus transmission as a function of *T*, *RH*, and *U*. In other words, it shows the potential strength of the virus depending on the weather conditions. We propose that AIR is a weather-dependent transmission rate parameter, which must be incorporated in epidemiological prediction models.

Given the above, this study aims at

• Linking the effects of weather with epidemiological predictions.
• Defining a new airborne infection rate index. Demonstrating the seasonality effects through specific examples worldwide.
• Showing that two pandemic waves are a natural phenomenon during the spread of disease linked to what we call weather seasonality.
• Establishing a connection between multiphase fluid dynamics with epidemiology.

This study does not aims at

• Proposing a generic model for infectious diseases, including references to any known pathogens.
• Improving the most recent version of SIR-derived models. One can use the weather effects introduced in this paper in conjunction with any other version of the SIR (or other) model. We emphasize that any model can only mimic the virus spread since it encompasses several assumptions about transmission, disease, and immunity.
• Producing results for every worldwide city. We could do that because we have available weather data, but we aimed to selectively present data, as an example, for some cities worldwide.
• Addressing issues of the host–pathogen interaction, which are not taken into consideration by many other models. It is beyond the scope of this study to discuss how microbes, germs, and pathogens (viruses, bacteria, and parasites) sustain themselves within host organisms on a molecular, cellular, organismal, or population level.
• Investigating other factors such as the behavior of the people in the spread of the disease.

## II. A NEW WEATHER-DEPENDENT TRANSMISSION RATE

### A. Virus concentration rate and weather conditions

The viability of airborne virus constitutes an essential indicator for the transmission rate during a pandemic. Scientists have tried to quantify the effects of temperature and relative humidity on the viability of airborne viruses.^18–21^ Virus infectivity^22^ depends on the virus particle structure in lipids (virion or capsid nanostructure). It is noteworthy that our knowledge about the virus structure is limited. Recently, Kanso *et al.*^14^ showed that the virus structure governs its rotational diffusivity. Specifically, using the general rigid bead–rod theory^23^ (and references therein), they showed that the virus rotational diffusivity descends monotonically with its number of peplomers. The recent study by Kanso *et al.*^14^ opens exciting possibilities for future research relating to the structure of CoV and its associated modeling.

**FIG. 1.**
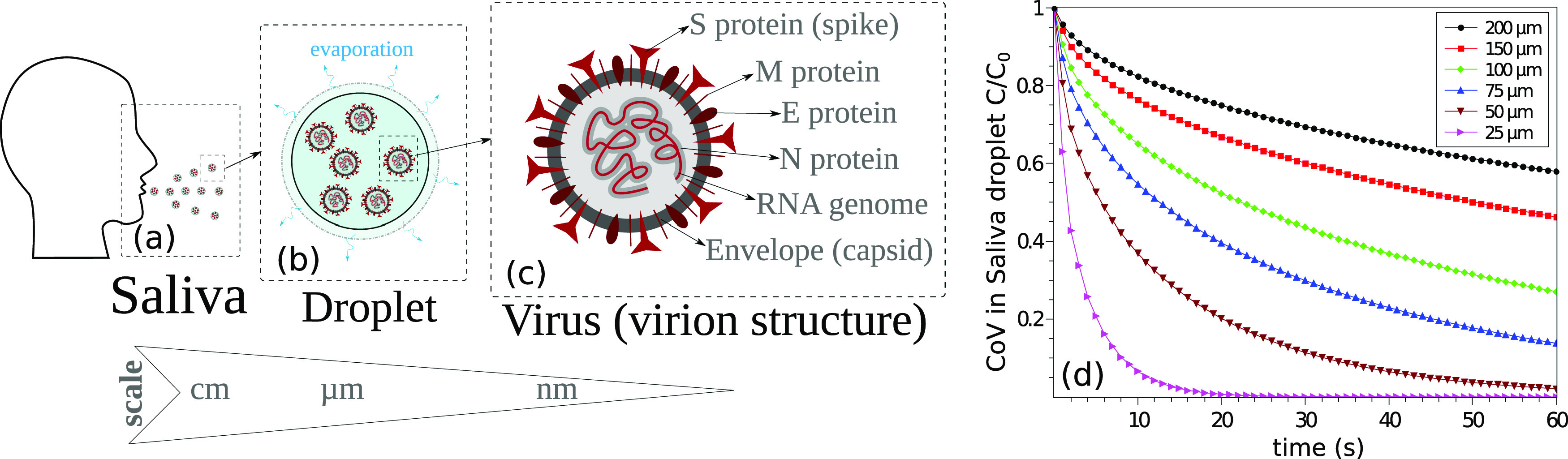
Climate effects on airborne virus transmission. (a) An infected individual expelling contaminated saliva droplets. (b) Coronavirus (CoV) particles in saliva droplets at an initial concentration *C*_0_, showing the evaporation process. (c) The structure of a CoV particle. (d) An example of the computed *C*/*C*_0_ variation with time at environmental conditions of 4 km/h wind speed, T = 20 °C, and RH = 50%.

In the COVID-19 pandemic, Coronavirus (CoV) is detected with high fidelity in the saliva of infected persons.^24^ Thus, the saliva has a very high potential for the diagnostic and transmission of COVID-19 among humans.^25–28^ Saliva droplets are expelled from the mouth or nose of an infected individual at an initial CoV concentration denoted *C* = *C*_*CoV*_ (Fig. 1). The contaminated saliva droplets are projected onto the surrounding environment that has a temperature (T), relative humidity (RH), and wind speed (U). Then, transport, evaporation, and settling of the droplets occur as natural processes such that the droplets settle down to the ground or suspend in the surrounding air. During these processes, the concentration *C* reduces with time at different rates as a function of T, RH, and U.

Using multiphase Computational Fluid Dynamics (CFD), we computed the variation in *C*_*CoV*_ with time for a wide range of weather conditions (0 °C ≤ *T* ≤ 40 °C, (10% ≤ *RH* ≤ 90%), and 4 km/h ≤ *U* ≤ 20 km/h). The advanced Eulerian–Lagrangian CFD model developed in Refs. 10, 11, and 17 was extended to account for virus concentration in droplets and its evolution under different weather conditions.

The reader can find the computational models in a previous study.^11^ Here, we extended the models to account for the concentration variation in saliva and predicted the evolution of airborne virus concentration in expelled saliva droplets under different environmental conditions. From hundreds of CFD simulations, we developed a reduced-order model (ROM) as an innovative virus airborne infection rate (AIR) index that is directly proportional to the virus concentration rate (CR). AIR is employed to quantify the potential of airborne coronavirus survival under different climate conditions (average temperature, relative humidity, and wind speed) in several worldwide cities.

To predict the evaporation of an airborne liquid saliva droplet containing virus particles, we take into account the structure and thermal properties of the virus particle as effective thermal properties of the saliva–virus mixture in the droplet [Figs. 1(b) and 1(c)]. As shown in Fig. 1(c), CoV is a roughly spherical particle with diameter *d*_v_ ranging from ∼50 nm to 150 nm with a mean diameter of 100 nm, made of RNA, four main proteins:^29,30^ Spikes (S) glycoproteins (“coron” form), Envelope (E) proteins, Membrane (M) proteins, and a single-stranded RNA genome (the genetic code) covered by a nucleocapsid (N) phosphoprotein, and a capsid or virion, which is made of a phospholipid bi-layer^31^ and protects the RNA.

**FIG. 2.**
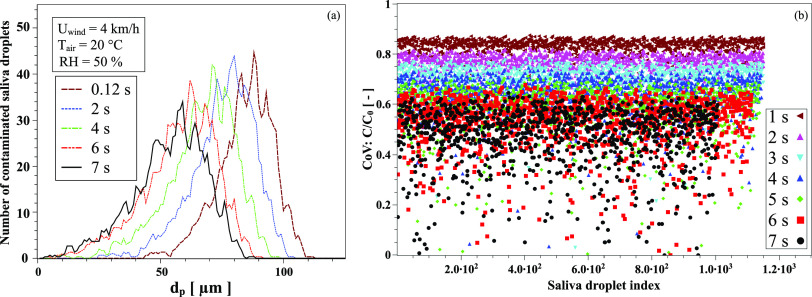
An example of the climate conditions’ effect on airborne virus transmission for the case study at 4 km/h wind speed, T = 20 °C, and RH = 50%. (a) Size distribution of contaminated saliva droplets emitted by a cough from an infected person; droplets expelled initially into the environment at a cough speed of about 8.5 km/h and own a non-uniform droplets size distribution.^10^ (b) Concentration of CoV in *each* expelled saliva droplet; assuming initially a uniform distribution of virus particles over the droplets population, the CoV concentration decreases in each droplet as function of time at different proportions and different rates.

Figure 1(d) shows the CoV dimensionless concentration as a function of time. It represents a concentration change associated with the reduction in the saliva droplet diameter due to the evaporation process and the internal diffusion of CoV particles. The concentration inside the droplet is assumed to be homogeneous. The internal convection is considered to have a negligible effect due to the small sizes of the saliva droplet (droplet diameter *d*_*p*_ < 300 *µ*m). An example of the saliva droplet size distribution is shown in Fig. 2(a) at *T* = 20 °C, *RH* = 50%, and *U*_W*ind*_ = 4 km/h. Figure 2(b) illustrates the concentration of virus in *each* expelled saliva droplet. For an initial uniform distribution of virus particles, the CoV concentration, *C*, decreases in each droplet as a function of time at different proportions and different rates,

$$C/C_{0}\;=\;e^{-7.5\sqrt{D_{v}\left(t\right)\;t}/d_{p}\left(t\right)}.$$ (1)

*d*_*p*_(*t*) is the saliva droplet diameter that changes with time depending on the evaporation rate and the saliva droplet size distribution. *D*_v_(*t*) is the time dependent diffusion coefficient of a virus particle in a saliva droplet given by *D*_v_(*t*) = *k*_*B*_*T*(*t*)/3*πμ*(*t*)*d*_v_. *d*_v_ is the virus capsid external mean diameter (*d*_v_ = 100 nm), *T*(*t*) is the time-dependent effective temperature of the saliva droplet, *k*_*B*_ is the Lattice Boltzmann constant, and *μ*(*t*) is the time-dependent liquid saliva viscosity.

**FIG. 3.**
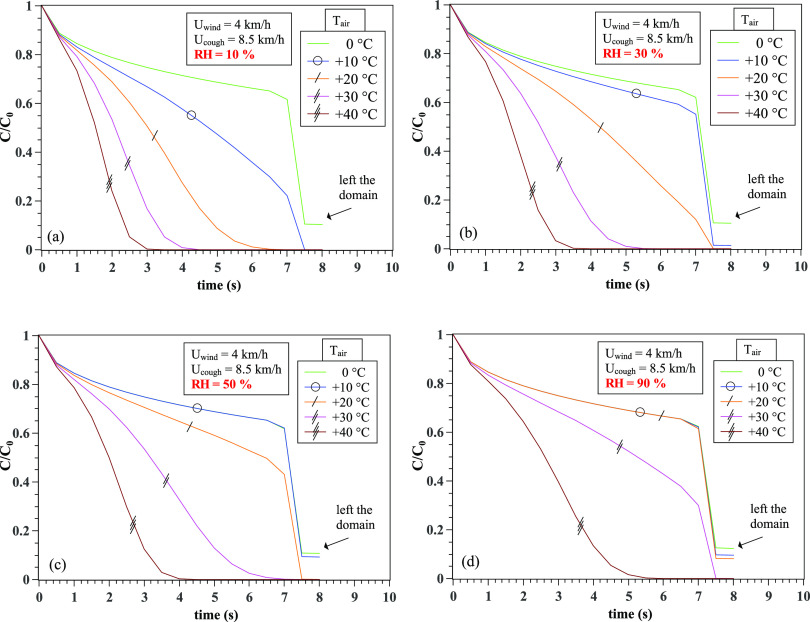
The combined effect of relative humidity and air temperature on the CoV concentration in saliva. An example at 4 km/h wind speed and relative humidities (a) RH = 10%, (b) RH = 30%, (c) RH = 50%, and (d) RH = 90%. The sharp decrease in the values is not physical; it is related to the fact that the droplets’ cloud left the computational domain located at 8 m away from the mouth of an individual.

An example of the variation in *C*/*C*_0_ with time at different weather conditions (T, RH, and U) is shown in Fig. 3. Sharp reductions occur at the maximum extremity of the x axis. The above is due to the geometrical boundary limit of the computational domain, i.e., the droplets cloud has exited the computational field (located at 8 m away from the entry or the mouth of the individual).

**FIG. 4.**
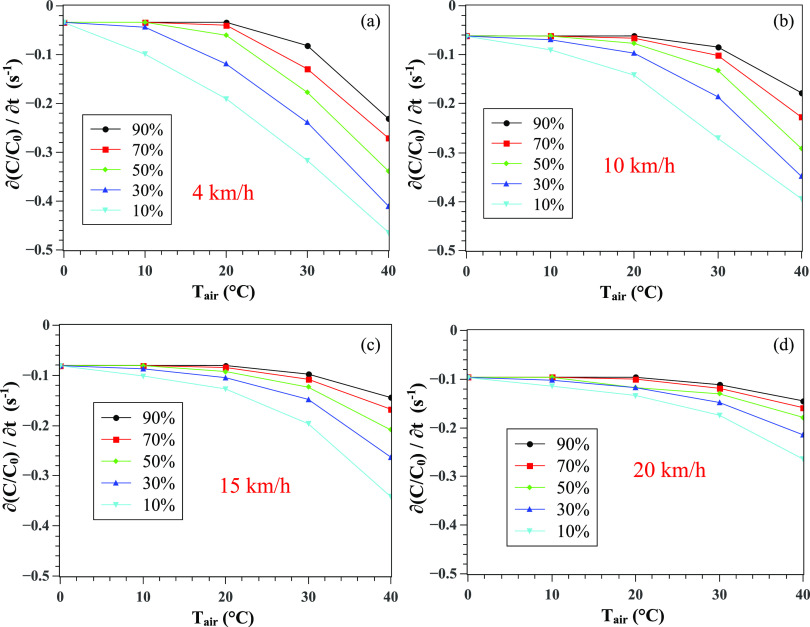
Effect of weather conditions on the CoV concentration rate (per unit time) as a function of the wind speed, temperature, and relative humidity. Wind at (a) 4 km/h, (b) 10 km/h, (c) 15 km/h, and (d) 20 km/h. The concentration *C* is made dimensionless by division by the initial concentration *C*_0_ at *t* = 0.

To better quantify the physical meaning of the results in Fig. 3, we focus on computing the slope variation with time, i.e., (*∂C*/*C*_0_)/*∂t*, that is shown in Fig. 4 for different weather conditions. We varied the wind speed from 4 km/h to 20 km/h and performed the simulations for a range of temperatures and relative humidity. We observe monotonic changes in the CoV concentration. We have used the average wind speed recorded for the three cities we examined. Thus, the maximum speed we used is 20 km/h. The wind speed between buildings and in residential areas is also always less than in free open spaces. The proposed model we discuss in Sec. II B could be extended to cover for any range of weather parameters. To make all values positive for CR, we add 0.5 to the right side of [*∂*(*C*/*C*_0_)/*∂t*],

$$CR=\partial \left(C/C_{0}\right)/\partial t\;+0.5.$$ (2)

### B. Airborne infection rate

The CR is directly proportional to the virus survivability. Thus, it provides an appropriate indicator for the airborne transmission, which we introduce in this study as “Airborne Infection Rate (AIR),”

AIR=CR. (3)

In fact, 0.5 added to the right side of the [*∂*(*C*/*C*_0_)/*∂t*] term in Eq. (2) makes AIR positive and bounded between 0 and 0.5; thus, an appropriate indicator of the airborne virus survivability and airborne infection rate is justified as follows:

• When *CR* → 0 [*∂*(*C*/*C*_0_)/*∂t* → −0.5], there exists a high reduction rate of the CoV concentration in saliva droplets; thus, we consider the virus being either eliminated completely or being in a “weak state” (*AIR* → 0 implies a low airborne infection rate).
• When *CR* → 0.5 [*∂*(*C*/*C*_0_)/*∂t* → 0], then there exists a low reduction rate of the CoV concentration in saliva droplets; thus we consider the virus being either live or in a “strong state” (*AIR* → 0.5 implies a high airborne infection rate).

The CR values between 0 and 0.5 are bounded between 0 and 1 using the operator 〈*〉 that transforms a dimensional physical variable *ξ* into a dimensionless one denoted by ξ^*^ such that

$$\xi^{*}=\frac{\xi -min\left(\xi \right)}{max\left(\xi \right)-min\left(\xi \right)},$$ (4)

where *min* and *max* are the minimum and maximum values of *ξ*, respectively.

**FIG. 5.**
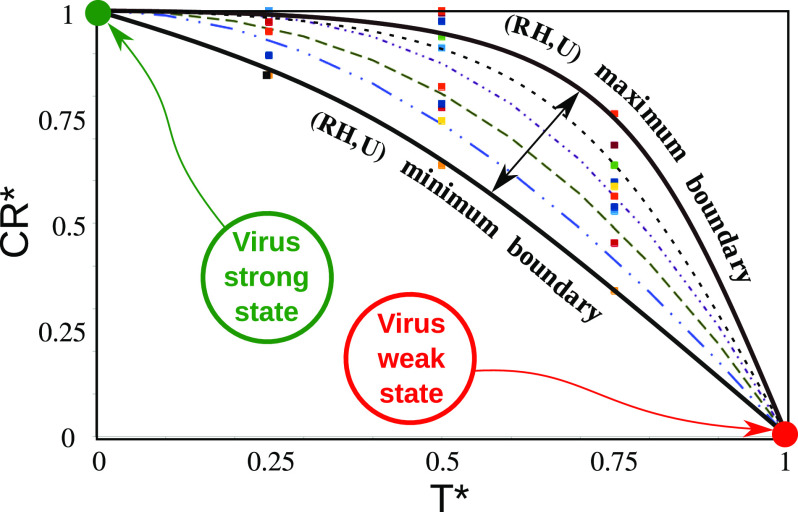
Scaling of the virus concentration rate (CR) with temperature (*T*), relative humidity (*RH*)). and wind speed (*U*). The solid square symbols represent the data points from the simulations. The lines (dashed, dotted-dashed, and solid) represent the single model predictions, which were found to fit all the data points very accurately. The two black solid lines are the maximum and minimum boundary limits for the range of data used to produce the results, (0 °C ≤ *T* ≤ 40 °C), (10% ≤ *RH* ≤ 90%), and (4 km/h ≤ *U* ≤ 20 km/h). The green and red circles show the strong and weak states of the virus particles, respectively. All the real variables denoted by *ξ* are made dimensionless by the 〈*〉 operator [see Eq. (4)]. *min*(*CR*) ≈ 0 and *max*(*CR*) ≈ 0.5.

The dimensionless quantities showed that the data points collapse into a zone bounded by two curved boundaries (black dotted-lines of Fig. 5) denoting the minimum and maximum values of RH and U. We found that the best model (the lines in Fig. 5) for the data points is

$$CR^{*}=F\cdot \left(RH^{*}+U^{*}\right)\cdot \sin^{*}\left(T^{*}\right)+\cos^{*}\left(T^{*}\right),$$ (5)

where F is given by

$$F=0.125\left(1-{\left(2T^{*}-1\right)}^{2}\right).$$ (6)

Note that CR^*^ can be transformed back into *CR* using Eq. (4) with *min*(*CR*) = 0, *max*(*CR*) = 0.5. The above equations clearly show that the model incorporates and will depend on the main weather parameters of temperature, relative humidity, and wind speed.

## III. WEATHER-DEPENDENT EPIDEMIOLOGICAL MODEL

The extensive high-fidelity simulations led to *CR* = *AIR* as a function of T, RH, and U. We consider AIR as a good indicator for airborne virus transmission and suggest it as a flow physics relevant parameter in epidemiological models.

As a physics-based simulation model, we consider a standard SIR model^8^ given by

$$\frac{dS}{dt}=-\beta \;S\;I/N,$$ (7)

$$\frac{dI}{dt}=\beta \;S\;I/N-\gamma I,$$ (8)

$$\frac{dR}{dt}=\gamma \;I,$$ (9)

where *β* = *AIR* is now a physics-based weather-dependent parameter and *γ* is the recovery rate coefficient that depends on the individual’s health and immunity system. *t* is time, and *N* is the population number. *S*, *I*, and *R* are the number of *susceptible*, *infected*, and *recovered* individuals, respectively. *β* represents the probability per unit time that a susceptible individual becomes infected. *γ* represents the probability per unit time that an infected person becomes recovered and immune.

The choice of the standard SIR model is based on the fact that it contains fewer parameters compared to other variants of the model in the literature. We believe that the advantage of using less complicated pandemic models reduces the uncertainty in forecasting. It is noteworthy that any model can only mimic the COVID-19 spread since it encompasses several assumptions about transmission, disease, and immunity.^32^

### A. Pandemic predictions

**FIG. 6.**
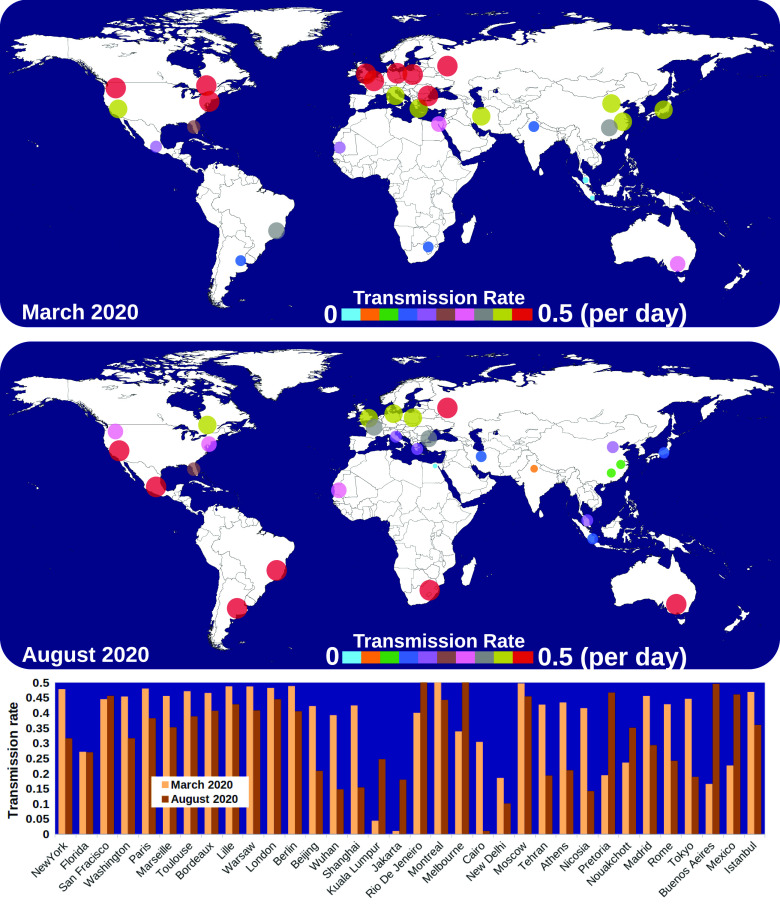
Weather-dependent transmission rate (*β*) in different cities worldwide during March and August 2020. The highest transmission, related to the CoV airborne concentration rate, is found to be about 0.5 per day. The above implies that the probability is *P* = 1 (100%) for a susceptible individual to be infected in two days due to the weather conditions (wind speed, temperature, and relative humidity) in different regions.

Considering that (*β*) is directly linked to *AIR*, we have computed an example of the transmission rate for different cities worldwide (Fig. 6). The results show that in March 2020, the airborne infection rate (transmission rate) increases as we move toward the northern regions of the planet. While in August 2020, it starts to grow in the southern areas.

Figure 5 presents a more in-depth scientific explanation for the relation between the CoV concentration rate (CR) and the weather conditions (temperature T, relative humidity RH, and wind speed U). The symbols represent the data points obtained from the numerous high-fidelity simulations. The dashed–dotted lines stand for the single model that fits all the data points accurately.

The two black solid lines show the maximum and minimum boundary limits for RH and U such that 10% ≤ *RH* ≤ 90% and 4 km/h ≤ *U* ≤ 20 km/h (the range of data used to produce the results). The green and red circles show the strong and weak states of the virus particles, respectively. At high temperatures and low virus concentration rates, the virus is in its weak state. At low temperatures and high virus concentration rates, the virus is in its strong state.

**FIG. 7.**
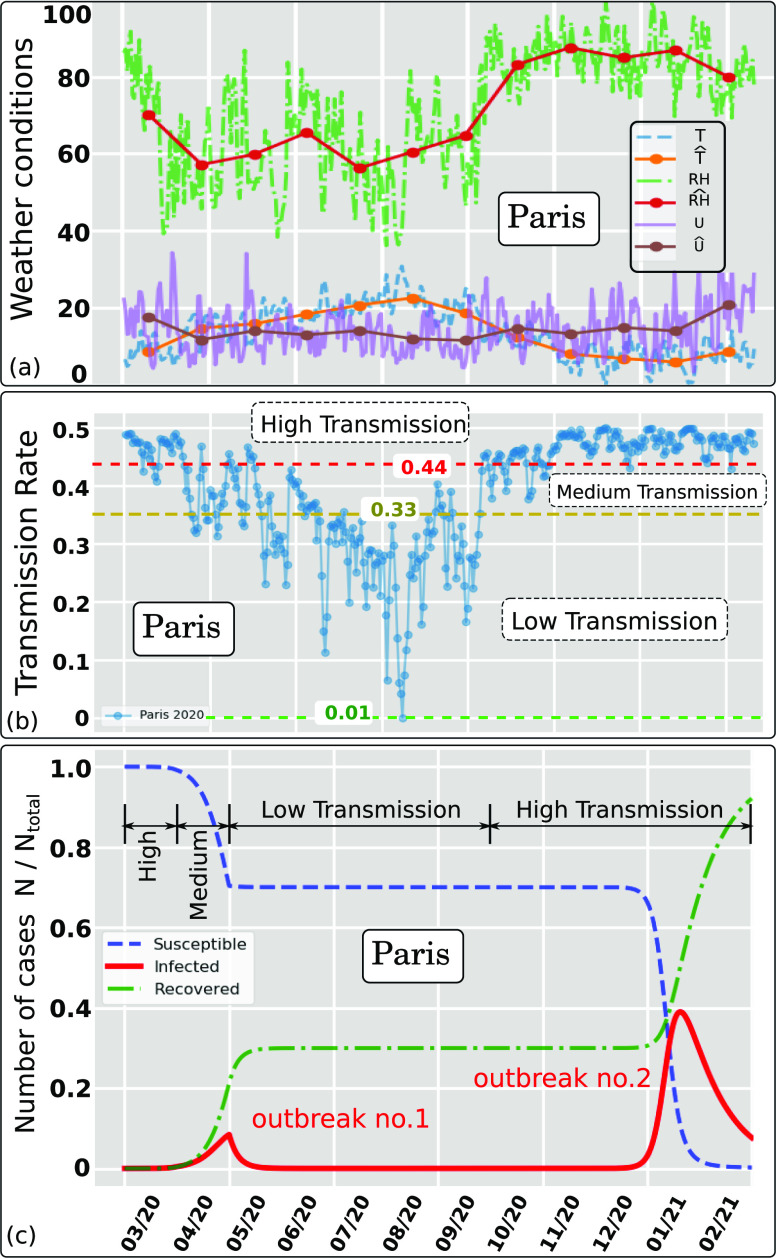
Effect of weather conditions (wind speed, temperature, and relative humidity) on the Airborne Infection Rate index (AIR = *β*) for Paris in 2020. The hat symbol denotes daily weather data averaged per month. (a) Weather data recorded between March and October 2020 (included) and estimated weather data between November 2020 and February 2021 based on the last year’s recorded weather. (b) Weather dependent transmission rate showing three trends denoted high, medium, and low separated by the respective threshold values 0.44 and 0.33. (c) Pandemic modeling and long time prediction (daily number of cases) using the weather-dependent transmission rate [Fig. 7(b)] in the standard SIR model.^8^ Two outbreaks predicted due to the weather seasonality in Paris ($\hat{i}$le de France) using *I* = 73 as total infected individuals in Paris on 1 March 2020 (source: WHO^1^). *N*_*total*_ ≈ 12.279 · 10^6^.

Using several high-resolution simulations, we computed *AIR* and inserted them into the standard SIR model. Figures 7(a) and 7(b) show in detail the effect of weather conditions on the transmission rate for Paris between March 2020 and February 2021. The weather history [Fig. 7(a)] was recorded between 1 March 2020 and 31 October 2020. The estimates are for the period between 1 November 2020 and 29 February 2021. Figure 7(b) shows clearly three different trends of the *AIR* index that describe different levels of airborne transmission; we divide them into high, medium, or low levels according to the changes in the weather seasonality. Two pandemic outbreaks are observed for Paris [Fig. 7(c)]. After the first outbreak, a considerable number of the population is still highly susceptible to infection. However, they were not infected due to the weakening of the virus during the summer period (virus weak state as shown in Fig. 5).

Note that constant transmission rate models cannot predict more than one pandemic outbreak over time. The second outbreak we predicted using a weather-dependent transmission rate (*β*) captures well the effect of seasonality. The model predicts a second pandemic outbreak in Paris to occur between January and February 2021 (strong virus state as shown in Fig. 7).

**FIG. 8.**
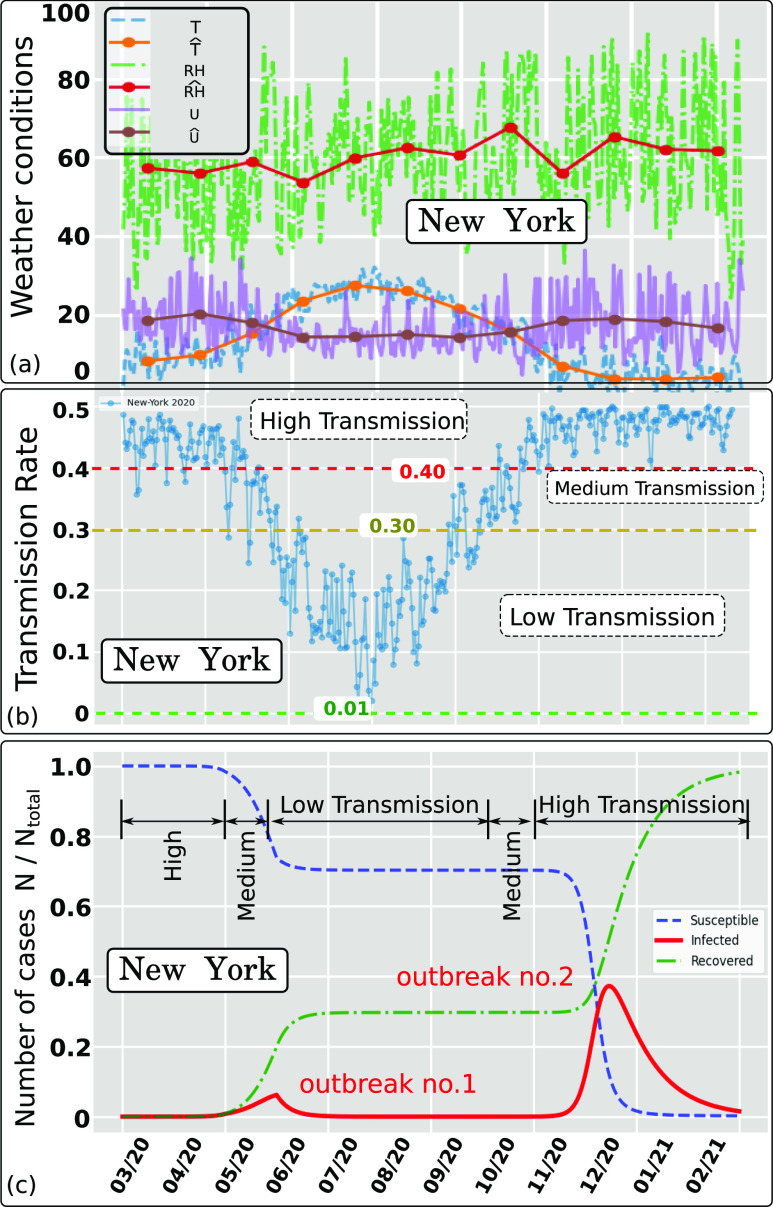
Effect of weather conditions (wind speed, temperature, and relative humidity) on the Airborne Infection Rate index (AIR = *β*) for New York state (USA) in 2020. The hat symbol denotes daily weather data averaged per month. (a) Weather data recorded between March and October 2020 (included) and estimated weather data between November 2020 and February 2021 based on the last year’s recorded weather. (b) Weather dependent transmission rate showing three trends denoted high, medium, and low separated by the respective threshold values 0.40 and 0.30. (c) Pandemic modeling and long time prediction (daily number of cases) using the weather-dependent transmission rate [Fig. 8(b)] in the standard SIR model.^8^ Two outbreaks predicted due to the weather seasonality in New York using *I* = 1 as approximate total infected individuals on 01 March 2020. *N*_*total*_ ≈ 19.47 · 10^6^.

As an additional example, Figs. 8(a) and 8(b) show the effect of weather conditions on the transmission rate in New York between March 2020 and February 2021. The weather history in Fig. 8(a) was recorded for New York between 01 March 2020 and 31 October 2020. The estimates are for the period 1 November 2020 to 29 February 2021. Figure 8(b) illustrates three different trends of the *AIR* index that describe different levels of airborne transmission in New York; we divide them into high, medium, or low levels according to the changes in the weather seasonality. Similar to Paris, two pandemic outbreaks are observed for New York [Fig. 7(c)], and a considerable number of the population is still highly susceptible to infection after the first outbreak.

**FIG. 9.**
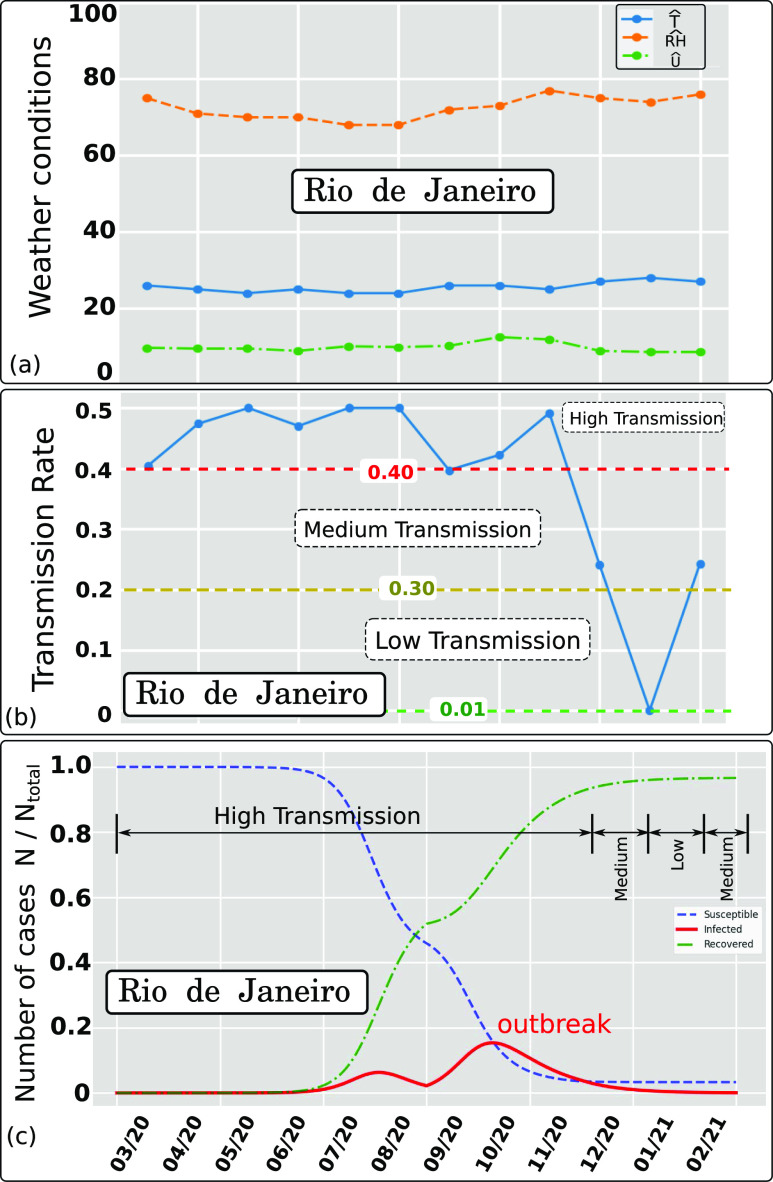
Effect of weather conditions (wind speed, temperature, and relative humidity) on the Airborne Infection Rate index (AIR = *β*) for Rio de Janeiro in 2020. The hat symbol denotes daily weather data averaged per month. (a) Weather data recorded between March and October 2020 (included) and estimated weather data between November 2020 and February 2021 based on the last year’s recorded weather. (b) Weather dependent transmission rate showing three trends denoted high, medium, and low separated by the respective threshold values 0.40 and 0.30. (c) Pandemic modeling and long time prediction (daily number of cases) using the weather-dependent transmission rate [Fig. 9(b)] in the standard SIR model.^8^ An outbreak predicted due to the weather seasonality in Rio de Janeiro using *I* = 1 as approximate total infected individuals on 1 March 2020. *N*_*total*_ ≈ 13.458 · 10^6^.

We further investigate the sensibility of the model to the weather seasonality for the case of Rio de Janeiro (Fig. 9). Rio de Janeiro has a different weather seasonality (shifted in time) compared to New York and Paris. The above results in a different AIR index [Fig. 9(b)]. When we feed *AIR* into the SIR model, we predict two pandemic outbreaks in Rio de Janeiro (Brazil). They appear to have the shape of one episode between early June and the end of December (less sharp wave peaks compared to the two waves observed for Paris and New York). The above shows the sensitivity of the pandemic model to different weather seasonality in various regions around the globe.

## IV. CONCLUSION

We established a new relationship between weather seasonality and airborne virus transmission and introduced it to the SIR epidemiological model. The new model predicts pandemic outbreaks in connection with main weather parameters: temperature, wind speed, and relative humidity. The significant findings include the following:

• Weather plays an important role in the pandemic outbreaks. Therefore, it must be included in epidemiological predictions.
• The Airborne Infection Rate (AIR) index defined in this study through the concentration rate (CR) provides a direct link between multiphase fluid dynamics and the spread of the disease.
• The results suggest that two pandemic outbreaks per year are more likely a natural phenomenon that is directly related to the weather seasonality during a pandemic evolution. The above puts in question large scale, strict lockdowns, but, indeed, the decisions for the above are associated with broader socio-economic issues.
• The social protective measures, as well as the aggressive testing and contact tracing, using electronic tracking devices, and foremost checking everybody at the points of entry into a country and strict quarantine rules in designated places, can slow down the spread of the disease but cannot stop a second wave.
• The proposed AIR index can be used in conjunction with any SIR or SIR-derived model.

## Data Availability

The data that support the findings of this study are available from the corresponding author upon reasonable request.
